# Large-scale analysis of expression signatures reveals hidden links among diverse cellular processes

**DOI:** 10.1186/1752-0509-5-87

**Published:** 2011-05-29

**Authors:** Steven X Ge

**Affiliations:** 1 Department of Mathematics and Statistics, South Dakota State University, Brookings, SD 57006, USA

## Abstract

**Background:**

Cells must respond to various perturbations using their limited available gene repertoires. In order to study how cells coordinate various responses, we conducted a comprehensive comparison of 1,186 gene expression signatures (gene lists) associated with various genetic and chemical perturbations.

**Results:**

We identified 7,419 statistically significant overlaps between various published gene lists. Most (80%) of the overlaps can be represented by a highly connected network, a "molecular signature map," that highlights the correlation of various expression signatures. By dissecting this network, we identified sub-networks that define clusters of gene sets related to common biological processes (cell cycle, immune response, etc). Examination of these sub-networks has confirmed relationships among various pathways and also generated new hypotheses. For example, our result suggests that glutamine deficiency might suppress cellular growth by inhibiting the MYC pathway. Interestingly, we also observed 1,369 significant overlaps between a set of genes upregulated by factor X and a set of genes downregulated by factor Y, suggesting a repressive interaction between X and Y factors.

**Conclusions:**

Our results suggest that molecular-level responses to diverse chemical and genetic perturbations are heavily interconnected in a modular fashion. Also, shared molecular pathways can be identified by comparing newly defined gene expression signatures with databases of previously published gene expression signatures.

## Background

With a limited number of genes, cells have to effectively coordinate their responses to diverse perturbations. Different stimuli could activate the same molecular pathways and thus induce overlapping sets of genes. A classic example is response to cold, drought and salt stress in plants [1]. Evoking an opposite response might be beneficial in other circumstances. The MYC pathway, for example, induces proliferative growth under favourable conditions, but is suppressed by many stresses such as inflammation [2]. Studying correlations between these diverse responses compliments in-depth investigations focused on cellular responses to individual stimuli and will enhance understanding of complex regulatory mechanisms.

There are many examples of the co-regulation of the same set of genes in different biological processes. For example, Chang *et al. *observed that the gene expression signature of serum response in fibroblast predicts cancer progression [3]. Similarly, diverse signaling pathways activated by growth factors induce broadly overlapping sets of genes [4]. Ben-Porath *et al. *found that genes over-expressed in histologically poorly differentiated tumors are enriched with genes highly expressed in embryonic stem cells [5]. On a larger scale, the Connectivity Map [6] provides a database of expression profiles of cultured cells treated with various compounds for the detection of associations of small molecules with similar mechanism of action. These studies are all based on the analyses of gene expression data and provide important insight into the relationship between different molecular pathways.

The objective of this study is to systematically compare published gene sets and create a "molecular signature map" that highlights correlations between diverse cellular perturbations. Published gene lists, however, are not readily available in a single source; they currently exist in scattered journal articles in diverse formats. The painstaking task of extracting this information manually has been attempted [7-10]. The L2L database represents the first systematic effort to collect lists of differentially expressed genes from microarray studies, which currently includes about 958 mammalian gene sets [8]. Oncomine is a web-based database system that focuses on cancer related genomics data and includes both raw microarray data and gene sets (referred to as "molecular concepts") [9]. The Molecular Signatures Database (MSigDB) was constructed as a knowledgebase for the popular pathway analysis program known as Gene Set Enrichment Analysis (GSEA) [10]. Most of the L2L information is included in MSigDB, which is by far the most comprehensive source of published human gene sets.

Furthermore, several tools to analyze gene lists data have been developed. Both the L2L and MSigDB web sites provide user interfaces to detect significant overlap of gene lists with their database. A similar approach, known as molecular concept analysis, is available at the Oncomine web site. In addition to using published gene sets, users can also compare their lists against functional gene sets, such as those derived from Gene Ontology (GO), KEGG, etc. Such analyses will broaden understanding of gene sets and their relationships with various pathways and functional categories.

This work is an effort to study the whole picture of overlapping gene lists. This comprehensive analysis of MSigDB gene sets related to chemical and genetic perturbations will provide insights on the relationship of diverse cellular processes. By representing overlaps between gene sets as networks, we focus on the interpretation of the connections among diverse gene sets by taking advantage of the methods for visualizing and analyzing complex biological networks.

## Results

### Thousands of significant overlaps are identified

The Version 2.5 of MSigDB contains 1,186 gene sets in the "C2: chemical and genetic perturbations" category [10], manually compiled from over 300 publications. It represents an important source of accumulated knowledge of the molecular signatures of various genetic and chemical perturbations. Except for about 99 gene sets that are based on mouse studies, most of the sets are derived from studies using human tissues or cells. The total number of distinct genes across gene sets in all publications is 14,553. Each gene set has a name like "COLLER_MYC_DN," where Coller is the first author of the publication [11] followed by a brief description of the set, such as "Genes down-regulated by MYC in 293T (transformed fetal renal cell)."

The 1,186 gene sets have a median size of 42, but vary greatly from 3 to 1,838 genes. Interestingly, the distribution is close to normal on a log scale (Additional File 1: Figure S1). Some of the most frequently appearing genes in these gene sets are cytokines and growth factors (Table 1). As suggested by the number of PubMed records associated with each of the genes, most of the top genes have been studied extensively (Table 1). MYC, STAT1, and ID2 are the three most common genes in published gene sets in MSigDB. Interestingly, the transcriptional repressor ID2 (inhibitor of DNA binding 2) is frequently identified as differentially expressed, even though it has been investigated in relatively few studies.

**Table 1:** Top 20 most frequently appearing genes in 1,186 published gene sets in MSigDB

Gene Symbol	Frequency in MSigDB C2	#PubMed Records	Gene Symbol	Frequency in MSigDB C2	#PubMed Records
MYC	86	13323	TNFAIP3	54	201
STAT1	75	2719	CDC2	54	4052
ID2	66	364	IL8	52	675
CDKN1A	64	6713	HMGB2	52	151
IFITM1	62	41	RHOB	50	270
SERPINE1	59	6486	GADD45A	50	224
ISG15	58	192	PCNA	49	8179
VEGF	56	22283	CCND1	49	7243
CTGF	56	1024	ATF3	49	207
IL6	55	2100	TOP2A	48	136

We carried out a comprehensive all-vs.-all comparison of the 1,186 published gene sets using a Perl script (available as Additional File 2). Based on the hypergeometric distribution, we then calculated the likelihood of observing the number of overlapping genes if these two gene sets are randomly drawn without replacement from a collection of 14,553 genes.

Using the Bonferroni correction for multiple testing, we multiplied P values by the total number of comparisons. After correction, the number of significant overlaps is 2,441. Some extremely significant (P < 1 × 10^-200^) overlaps are apparently justified by the biology. For example, 120 out of the 149 genes in the gene set "CHANG_SERUM_RESPONSE_UP" are shared with "SERUM_FIBROBLAST_CORE_UP", which only has 205 genes. Therefore, even with the most conservative correction, thousands of significant overlaps can be identified.

Since the Bonferroni correction could be too conservative, we used the false discovery rate (FDR) procedure [12] in further analysis. Although the tests are not statistically independent due to the overlaps between sets, the dependency should be considered a positive correlation, and the FDR procedure is applicable [13]. The raw P-values were translated into FDR to correct for multiple testing [12]. Overlaps between gene sets from the same study were considered trivial and were removed. With FDR < 0.001 as a cut-off, we identified 7419 significant overlaps between 958 gene sets.

To further validate the significance of these overlaps, we used the same criteria to detect overlaps from data generated under the null hypothesis. We generated 1,186 gene sets of the same sizes as those in MSigDB but with genes drawn randomly from a pool of 14,553 distinct genes. With FDR < 0.001 as the cut-off, no significant overlap was identified. The same results hold in five repeated simulations. This simulation demonstrated the significance of the 7,419 overlaps in MSigDB.

### Modular organization of the gene set overlapping network

**Figure 1:**
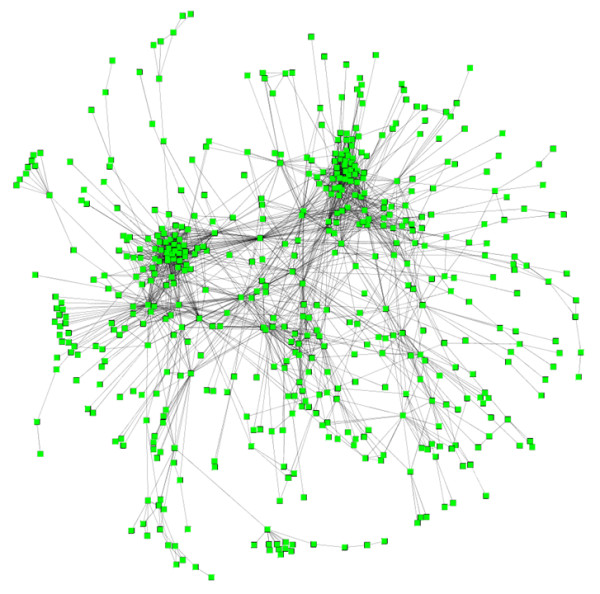
**A highly connected network of published gene sets**. Nodes represent gene sets and edges represent significant overlaps. We identified 7,419 significant overlaps (FDR < 0.001) among 958 published gene sets. Only 2,915 highly significant (FDR < 1.0 × 10^-6^) overlaps are shown in this figure. We observed a large number of significant overlaps among diverse expression signatures organized in a modular fashion.

Our results can be conveniently represented by an undirected network, where nodes correspond to gene sets and edges indicate significant overlaps (Figure 1). An annotated version of this network with detailed information on gene sets and overlaps can be found in Additional File 3. This file can be read by the Cytoscape software (http://www.cytoscape.org) [14] for easy access and exploration. This same information is also provided as an Excel file (Additional File 4). This network highlights correlations across expression signatures of diverse biological processes, diseases, and cellular stimuli. This big network thus constitutes a "molecular signature map," in which individual perturbations are placed in the context defined by all others.

This is a highly connected network with an average of 7.74 connections per gene set. Surprisingly, most (949) of the 958 gene sets are connected to a dominant main network. In this network, while most nodes are connected to a small number of other gene sets, there are a small number of gene sets that significantly overlap with a large number of gene sets. This is similar to what has been observed in many biological networks.

One noticeable feature of the molecular signature map in Figure 1 is its modularity. We observed several clusters of highly connected expression signatures. An efficient way to organize a large number of responses to diverse perturbations is to organize these responses into modules. Figure 1 supports the notion that cells coordinate their responses to various stimuli by the combination of multiple modules.

To identify these modules, or highly interconnected sub-networks, we used the MCODE algorithm [15] to analyze the network of 949 nodes. We identified 21 sub-networks with four nodes or more. The biggest sub-network was further partitioned into two due to its size and topology. Thus, we obtained a total of 22 sub-networks. Table 2 lists these sub-networks with detailed information on both biological themes and the most frequent genes. These are the modules that cells use to remain viable in diverse environments.

**Table 2:** Summary of 22 modules consisting of groups of heavily interconnected gene sets

ID	#Sets	Cluster Density	Representitive GeneSet	Biological Theme	Most freqently shared genes between gene sets	Most significantly enriched GO Term	P values
1a	31	13.5	P21_P53_ANY_DN_49 (Sup. Figure 1)	Cell cycle, especially M phase	FOXM1,CCNB1,KIF2C,KIF11,CDC2,CCNB2,UBE2C,CDC20,MKI67,DLG7	Cell Cycle	2.80E-45
1b	29	12.8	DER_IFNB_UP_93 (Figure 3)	Immune response/interferon	MX2,MX1,OAS1,STAT1,OAS2,ISG15,IFITM1,IFIT3,IRF7,IFI27Response to virus		1.30E-19
2	15	3.7	IL1_CORNEA_UP_63	Inflammatory response/IL1	IL1B,CCL4,PLAUR,CXCL2,CCL5,TNFAIP3,CD44,IER3,NFKBIA,PLEK	Immune system process	1.00E-15
3	16	3.6	TNFA_NFKB_DEP_UP_18 (Figure 5)	Inflammatory response/TNFa related	GPNMB,CXCL1,SOD2,MMP9,CXCL5,CCL2,CHI3L1,CXCL3,CD9,IL1B	Immune system process	2.70E-20
4	14	3.1	GENOTOXINS_ALL_4HRS_REG_27 (Figure 4)	Cell cycle, DNA damage response	CKS2,ECT2,MAD2L1,BUB1,AURKA,CCNB2,CKS1B,PRC1,TRIP13,RRM2	Cell Cycle	3.10E-22
5	20	2.1	HOHENKIRK_MONOCYTE_DEND_DN_122	Inflammatory response/blood cells	STAT1,RAB2,CXCL3,HSPH1,SFRS3,S100A4,S100A8,TOP2A,ACTR1B,ANXA1	inflammatory response	7.40E-04
6	14	2.0	TGFBETA_ALL_UP_80	Cell adhesion, differentiation	IGFBP3,COL6A3,THBS2,CSPG2,SERPINE1,COL1A2,COL3A1,COL6A1,LOX,TIMP1	Cell adhesion	9.60E-07
7	6	2.0	ESR_FIBROBLAST_DN_18	cell differentiation	GNPNAT1,LCK,NDRG1,SOX7,TAF1C,TP53I11,ZNF507,CEPT1,GABBR1,HGF	--	
8	35	2.0	HYPOXIA_REG_UP_38 (Figure 6)	Cell cycle arrest	POSTN,MTHFD1,MYC,TFRC,COL6A3,CSPG2,CTPS,SNRPA1,WEE1,ADORA2B	cell cycle arrest	8.30E-02
9	18	1.7	UVC_XPCS_4HR_DN_242	Down-regulated by UV, TNFa	ITGB5,AXL,ARL4C,RGS4,ACTA2,ARHGAP1,ARL6IP5,CAP2,COL1A2,CYFIP2	Signal Transduction	7.50E-04
10	7	1.7	SCHUMACHER_MYC_UP_54 ( Figure 2)	MYC target genes	NME1,HSPE1,HSPD1,LDHA,TFRC,APEX1,CDK4,EBNA1BP2,ENO1,FKBP4	Intracellular organelle lumen	1.80E-02
11	5	1.6	GNATENKO_PLATELET_UP_47	Platelet genes	PF4,PPBP,TMSB4X,GPX1,HIST2H2AA4,ACTB,B2M,CCL5,CD99,CFL1	*	
12	7	1.6	ADIPOCYTE_PPARG_UP_16	Lipid metabolic process	ADIPOQ,AQP7,DGAT1,FASN,RETN,CIDEC,COX7B,NDUFS1,NR1H3,SCARB1	Lipid metabolic process	7.10E-04
13	27	1.6	CHANG_SERUM_RESPONSE_DN_194		SC4MOL,ACTB,HMGCS1,BAK1,ZYX,SCD,BAD,CTNNA1,FDPS,ITGB4	hemopoiesis	3.60E-03
14	4	1.5	CISPLATIN_PROBCELL_UP_17		ABI1,CDKN1A,EI24,LPIN1,TOB1,TP53INP1,TXNIP,ABLIM1,CARHSP1,H2AFJ	*	
15	6	1.3	UVC_LOW_ALL_UP_19	Response to DNA damage, UV	BTG2,CDKN1A,GDF15,BTG1,DDB2,PLXNB2,FDXR,GPRC5A	*	
16	7	1.3	FLECHNER_KIDNEY_TRANSPLANT_WELL_UP_565		RAB1A,ATP2A2,COL1A2,PGK1,RAB2,SPARC,WEE1,AGL,CFLAR,DNAJA1	*	
17	5	1.2	CMV_HCMV_TIMECOURSE_ALL_UP_470	IL6 induced	MX1,IRF4,TNFSF10,CCNC,DNAJB9,GATM,GNA13,HBEGF,ICAM1,JUNB	Response to stimulus	4.80E-02
18	5	1.2	REN_E2F1_TARGETS_50	E2F1 target genes	PCNA,TOP2A,KIAA0101,POLA2,RFC4	*	
19	6	1.2	STEMCELL_HEMATOPOIETIC_UP_1452	Stem cell enriched	ATP5D,BTBD14A,EEF1A1,GLUL,NTAN1,NUDCD2,PCGF2,ATP1A1,CD151,DYNC1H	*	
20	4	1.0	ZHAN_MULTIPLE_MYELOMA_VS_NORMAL_DN_4	Leukemia related	ELA2,BLNK,BZRAP1,CD24,CD7,CEBPD,CST7,DNTT,HGF,KCNE1L	*	
21	4	1.0	NADLER_OBESITY_DN_38	Obesity down, adipocyte up	PPA1,ACSL1,AGT,ALDH2,CFD,CRAT,FABP4,LDHB,PC,PPARG	*	

Cluster density is defined by dividing the number of significant overlaps (edges) by the number of gene sets within the sub-network.

* Less than 20 genes.

Many of these highly connected sub-networks reveal clusters of gene sets derived from biologically similar perturbations. This is evident from the coherent GO terms enriched in genes shared by gene sets within sub-networks (Table 2). We extracted 70 most frequently appearing genes in each sub-network and conducted enrichment analyses based on GO terms. See Additional File 5 for the full list of these top genes in each module.

Some unexpected links reveal interesting similarities in cellular responses to very different stimuli. We will discuss several of these sub-networks in the following sections. Additional sub-networks are discussed in Additional File 1. For each sub-network, we examine one or more examples of overlapping gene sets in details. These examples are summarized in Table 3.

**Table 3:** A summary of overlaps that were discussed in details in this paper

Overlapping Gene Sets	Explanation and supporting references	Repressive?
Chang_Serum_Response_up & Schumacher_MYC_up	The c-Myc oncogene mediates response to serum stimulation and triggers proliferative growth [18,19].	

Sana_IFNG_Endothelial_Dn & Zeller_MYC_Up, MYC_Targets	Interferon γ (IFNG) inhibits cell growth through suppression of c-MYC expression [2].	Yes

Taketa_NUP9_HOXA9_3d_Up and interferon α and β gene sets	Transduction of fusion protein NUP98-HOXA9 induces "up-regulation of IFNβ1 and is accompanied by marked up-regulation of IFN-induced genes" [20].	

CMV (cytomegalovirus) infection & Various cytokine regulated gene sets	Host cell response to CMV infection might be mediated by these cytokines.	

StemCell_Embryonic_up & BRCA_Prognosis_Neg	Aggressive tumors share some expression signature of embryonic stem cells [5].	

P53_Genes_All & Zeller_MYC_Dn	p53 represses the oncogene MYC possibly through miRNA-145 [45].	Yes

Gay_YY1_up & P53_Genes_All	YY1 inhibits the activation of p53 [36].	Yes

Cancer_undifferentiated_Meta_up & IDX_TSA_UP_Cluster3	Genes involved in TSA-induced differentiation of fibroblasts into adipocytes are also upregulated in undifferentiated tumors.	

Peng_Glutamine_Dn & several MYC upregulated gene sets	Glutamine starvation might suppress cell growth by repression of MYC pathway.	Yes

Manalo_hypoxia_Dn, StemCell_Embryonic_up, Le_Myelin_up	Cell cycle genes are regulated by hypoxia, stem cells, and growth after wounding.	Partly

### c-MYC oncoprotein and its relationships to serum stimulation and interferon γ

**Figure 2:**
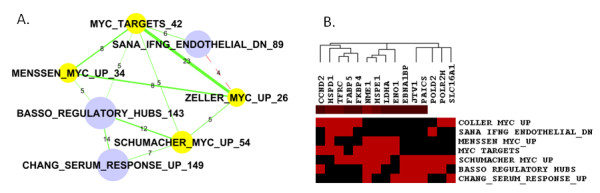
**Sub-network #10: MYC target genes**. A) The sub-network of MYC target genes. Nodes represent gene sets and edges represent significant overlaps. The size of the node indicates the number of genes in the gene set, which is also numerically labelled by the number at the end of each gene set name. The thickness of the edges is proportional to significance (-log10(FDR)). The labels of edges indicate the number of overlapping genes. Red dashed edges represent repressive connections between "X" upregulated genes and "Y" downregulated genes. The same settings were used to generate subsequent figures in this paper. Gene sets explicitly related to MYC oncoprotein are highlighted in yellow. B) List of 15 genes (columns) that appear three times or more in these seven gene sets (rows). Red indicates that a gene is included in a gene set. This figure shows that our approach can identify overlaps between biologically related gene signatures.

Some of the sub-networks confirm the overlapping of genes in studies investigating similar perturbations. One example is shown in Figure 2A (corresponding to Cluster #10 in Table 2). Four of the seven gene sets in this sub-network are clearly marked as target genes or are upregulated by oncoprotein c-Myc. The gene set of Basso *et al. *deals with hubs in gene regulatory networks; MYC is identified as a major hub [16]. Basso *et al. *also noticed that a significant proportion of MYC target genes are regulatory hubs [16]. Therefore, not surprisingly, the regulatory hubs are enriched with MYC targets. Significant overlaps between these five studies of MYC related genes are identified by our analysis, which reassures us that our analysis can identify biologically related gene sets.

This sub-network also highlights a gene set of serum response genes that overlaps with MYC gene sets [17]. The c-Myc oncogene is known to mediate responses to serum stimulation [18,19] and trigger proliferative growth in a favourable environment. The overlaps between two MYC target gene sets and genes downregulated by interferon γ (IFNG) were unexpected. However, as IFNG inhibits cell growth through suppression of c-MYC expression [2], upregulation of IFNG causes downregulation of MYC target genes. We could generalize that overlaps between a set of "X upregulated genes" with "Y downregulated genes" possibly indicate repressive interactions between factors X and Y. Such overlaps are highlighted in dashed red lines in the networks.

We conclude that most of the gene sets in this sub-network are directly or indirectly related to MYC protein. Figure 2B shows the list of 15 genes that appear three times or more in these seven gene sets. We think this could be a reliable list of MYC target genes based on multiple publications.

### A sub-network for pathogen response

**Figure 3:**
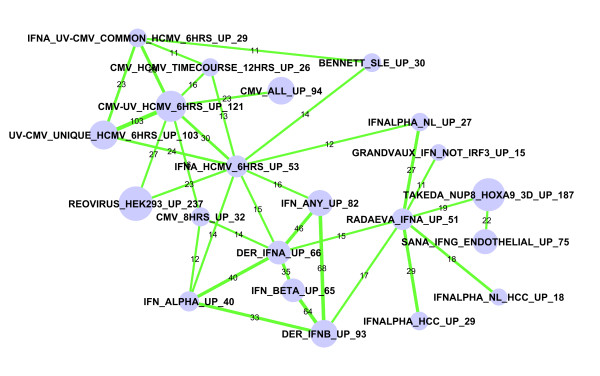
**Sub-network #1b: Expression signature of interferons α and β**. Only edges with extremely significant overlaps (FDR <1.0 × 10^-20^) are shown here. Nodes represent gene sets and edges represent significant overlaps. The size of the node indicates the number of genes in the gene set, which is also numerically labelled by the number at the end of each gene set name. The thickness of the edges is proportional to significance (-log10(FDR)). The labels of edges indicate the number of overlapping genes. This figure shows that expression signatures of interferon α and β are very similar across multiple studies and that pathogen responses rely on a core set of shared genes.

Another example of a similar perturbation is shown in Figure 3, which corresponds to sub-network #2 in Table 2. As this sub-network is so densely connected, only overlaps with extremely high significance (FDR < 1 × 10^-20^) are shown. Most of the 29 gene sets in this sub-network are regulated by interferons α and β that mediate response to pathogens. Overlaps among these sets are remarkably significant. For example, 35 out of the 66 DER_IFNA_UP genes are also included in the 65 genes in IFN_BETA_UP.

Correlations between several other gene sets and interferon α and β pathways are not obvious. The "TAKEDA_NUP9_HOXA9_3D_UP" gene set includes genes upregulated by the fusion protein NUP98-HOXA9, which occurs in acute myeloid leukemia [20]. Takeda *et al. *noted that transduction of this fusion protein induces "upregulation of IFNβ1 and is accompanied by marked upregulation of IFN-induced genes" [20]. Thus, their gene list must contain INFB target genes. The "BENNETT_SLE_UP" gene set includes genes significantly up-regulated by systemic lupus erythematosus (SLE) patients [21]. The major conclusion and surprising finding of this study are that the SLE active expression profile is "distinguished by a remarkably homogeneous gene expression pattern with overexpression of granulopoiesis-related and interferon (IFN)-induced genes" [21]. Finally, seven gene lists in this sub-network are related to CMV (cytomegalovirus) infection [22]. The finding that these gene sets are highly significantly related to IFN-induced genes indicates that host cell response to CMV infection might be mediated by these cytokines.

We further investigated whether the genes that are frequently shared by gene sets in this sub-network have coherent biological functions. The most significantly enriched functional category is "Response to virus" (P <1.3 × 10^-19 ^after Benjamini correction of multiple testing), followed by "Immune response" (P <1.5 × 10^-12^). Out of the 70 genes, 18 and 24 are associated with "Response to virus" and "Immune responses", respectively (Table 2). These results indicate that the gene lists in this sub-network are dominated by immune responses triggered by various conditions.

### Stem cell related genes as predictors of poor prognosis for breast cancer

**Figure 4:**
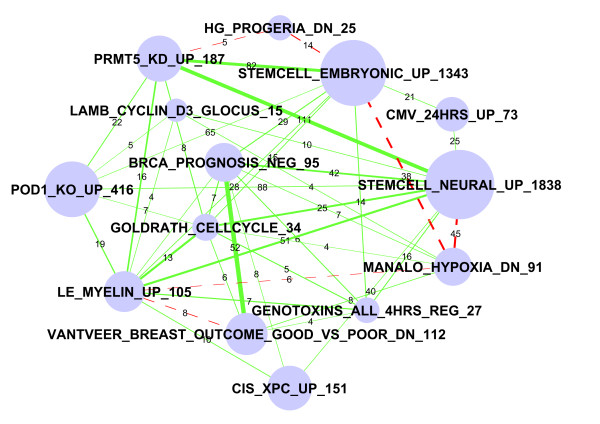
**Sub-network #4: Stem cell related gene sets and breast cancer prognosis predictors are linked**. Nodes represent gene sets and edges represent significant overlaps. The size of the node indicates the number of genes in the gene set, which is also numerically labelled by the number at the end of each gene set name. The thickness of the edges is proportional to significance (-log10(FDR)). The labels of edges indicate the number of overlapping genes. The labels of edges indicate the number of overlapping genes. Red dashed edges represent repressive connections between "X" upregulated genes and "Y" downregulated genes. We observed overlaps between stem cell related expression signature and breast cancer prognosis predictors. Expression of some cell cycle related genes is shared between stem cells and invasive breast cancers.

Sub-network #4 in Table 2 includes diverse gene sets (Figure 4). The "StemCell_neural_up" set includes 1,838 genes highly expressed in mouse neural stem cells, compared to differentiated brain and bone marrow cells [23]. Another related set from the same study is "StemCell_Embryonic_up" representing genes enriched in embryonic stem cells. Interestingly, these gene sets intersect significantly with genes associated with poor prognosis in breast cancers. The "BRCA_Prognosis_Neg" and "Vant Veer_Breast_Outcome_Good_vs_Poor_Dn" are derived from the same publication [24] and are extracted from the same list of genes whose higher expression predicts poor outcome. The latter includes many genes represented by clone IDs not properly mapped to gene symbols.

The overlap between stem cell and breast cancer prognosis genes is highly significant: 42 (44%) of the 95 genes in "BRCA_Prognosis_Neg" are highly expressed in embryonic stem cells ("StemCell_Embryonic_up", with FDR <1 × 10^-11^). These 42 genes are enriched with 11 cell cycle related genes (Benjamini corrected P < 0.00001), 20 of which are related to organelle parts of cell structure (Benjamini corrected P < 0.008). The significant overlap between breast cancer prognosis genes and stem cell genes thus highlights the similarity in expression profiles between aggressive tumors and stem cells. This is supported by a more in-depth meta-analysis of gene expression data [5].

Another interesting overlap is between stem cell gene lists with genes down-regulated by hypoxia. Thirty eight (42%) of the 91 genes in "Manalo_hypoxia_Dn" set are included in "Stem_Cell_Embryonic_up" with FDR <1 × 10^-12^. Of these 38 genes, 12 are related to GO Term "DNA replication" with Benjamini P value <8.5 × 10^-9^. Cell cycle genes are also enriched. One of the overlapped genes is BRCA1. Other lists in this cluster include "Genotoxins_All_4hrs_Reg," which is a list of genes that are commonly regulated by six types of genotoxins [25]. The overlapped genes are also mostly cell cycle related, including BUB1, CDC20, CCNB1, etc. The "Le_MYELIN_Up" set contains genes upregulated after sciatic nerve injury. Thus, these genes might be related to growth after wounding.

We also compared gene lists in this sub-network with sets of genes (NRC-1 to NRC-9) recently identified as breast cancer prognostic markers by Li *et al. *[26]. We identified modestly significant (unadjusted P value < 1 × 10^-4^) overlaps between three gene sets in this subnetwork with two gene sets related to cell cycle (NRC-1 and NRC-5) and one related to cell growth (NRC-9). See Additional File 1: Figure S3 for more information. These overlaps again suggest that cell cycle genes are important in predicting breast cancer survival. But further study is clearly needed to systematically compare the NRC and other breast cancer related gene sets, many of which are not included in the version 2.5 of MSigDB database.

### Glutamine starvation strongly downregulates MYC target genes

**Figure 5:**
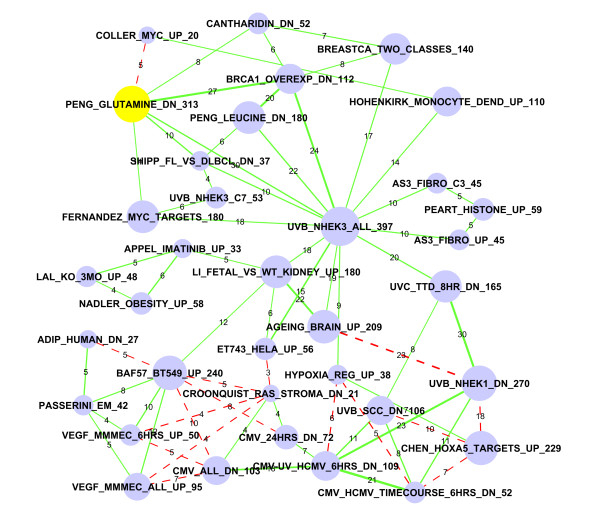
**Sub-network #8: Glutamine starvation and c-Myc genes**. The glutamine starvation down-regulated gene set is highlighted in yellow, which is connected to sets of MYC target genes. Nodes represent gene sets and edges represent significant overlaps. The size of the node indicates the number of genes in the gene set, which is also numerically labelled by the number at the end of each gene set name. The thickness of the edges is proportional to significance (-log10(FDR)). The labels of edges indicate the number of overlapping genes. The labels of edges indicate the number of overlapping genes. Red dashed edges represent repressive connections between "X" upregulated genes and "Y" downregulated genes. The "Peng_Glutamine_Dn" list significantly overlaps with almost all MYC related gene sets.

Sub-network #8 in Table 2 is shown in Figure 5, and includes diverse gene sets ranging from ultraviolet (UV) treatment, glutamine starvation, BRCA1 overexpression, etc. The connection between "Appel_Imatinib_up" and "Li_Fetal_vs_WT_kidney_up" confirms the regulation of differentiation by Imatinib, a new tyrosine kinase inhibitor. Imatinib can inhibit dendritic cell differentiation [27]. The "Li_Fetal_vs_WT_kidney_DN" gene set represents genes highly expressed in Wilms' tumor (WT) compared with fetal kidneys [28]. WT is characterized by arrested cellular differentiation. Even though the cells/tissues are different in these two studies, we were able to detect this moderately significant overlap.

We focus our attention on the "Peng_Glutamine_Dn" gene list that is associated with glutamine starvation in human BJAB B-lymphoma cells [29]. An unexpected connection is that genes downregulated by glutamine starvation contain many MYC target genes. In the whole network this is most evident as the "Peng_Glutamine_Dn" list significantly overlaps with almost all MYC related gene sets. The neighborhood of this gene set is in the molecular signature map in Additional File 1: Figure S2. Yuneva *et al. *showed that glutamine but not glucose starvation induces MYC-dependent apoptosis in human cancer cells [30], but the mechanism is unknown. On the other hand, Wise *et al. *found that overexpression of MYC promotes glutaminolysis and leads to cellular addiction to glutamine in cancer cells [31]. These study results may lead to the development of targeted killing of cancer cells that rely on high levels of glutamine uptake. We found no report on whether glutamine starvation inhibits the MYC pathway. If this is indeed true, as suggested by the overlapping of these gene sets, then the closely related nature of glutamine metabolism and the MYC pathway will need to be evaluated more closely.

To further confirm the link between glutamine deprivation and the MYC pathway, we downloaded and re-analyzed the raw DNA microarray data on glutamine starvation [29]. Using the GSEA program, we analyzed the whole dataset for enriched gene sets. The enriched gene sets are shown as Additional File 1: Table S1. One pathway that showed up is the proteosome degradation pathway, in which nutrient deficient cells suppress protein degradation as a means for survival. The most noticeable pathways are multiple MYC target gene sets downregulated at highly significant levels, confirming our observation based on gene set overlaps.

**Figure 6:**
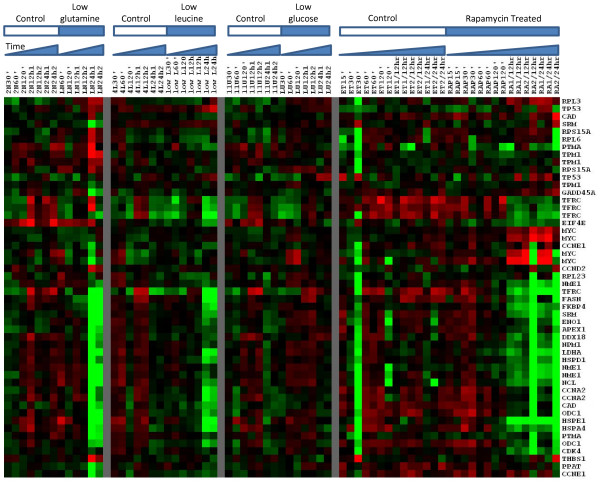
**Expression of c-Myc target genes in B-lymphoma cells upon glutamine, leucine and glucose starvation as well as rapamycin treatment**. Red and green denote higher and lower expression, respectively. Glutamine and leucine deficiencies, but not glucose deficiency, strongly downregulate many MYC target genes. The anticancer drug rapamycin has a similar effect on these genes, suggesting that rapamycin mimics amino acid starvation.

Figure 6 is a heatmap of relative expression levels of a list of 42 MYC target genes compiled from multiple studies of MYC transcriptional targets [32]. Glutamine and leucine deficiencies, but not glucose deficiency, strongly downregulate many MYC target genes. The anticancer drug rapamycin has a similar effect on these genes, suggesting that rapamycin mimics amino acid starvation. Downregulation is strongest after 24 hours of nutrient deficiency, or 12 hours after rapamycin treatment. Interestingly, glutamine and leucine starvation only lead to a modest decrease in MYC gene expression; rapamycin treatment even seems to upregulate its expression. This raises questions regarding the mechanism by which these target genes are downregulated. Some hints come from the well-studied effect of rapamycin. Rapamycin inhibits the TOR pathway, which regulates cell growth and cell cycle progression in many species. Rapamycin has been shown to downregulate MYC post-transcriptionally, by inhibiting mRNA translation [33]. Therefore, it is possible that glutamine starvation would have a similar course of action.

Glutamine starvation triggers a complex network of transcription factors including ATFs and C/EBP factors, and such response might be cell line- or species-dependent (See [34] for review). Indeed, our further analysis of another set of DNA microarray data [35] suggests that glutamine starvation does not cause downregulation of Myc target genes in mouse hepatoma cells (data not shown). However, for this specific B-lymphoma cell line studied by Peng *et al. *[29], the suppression of the MYC pathway is strongly supported by gene set overlaps and raw DNA microarray data analysis.

### Repressive interactions between pathways

Interestingly, we identified thousands of overlaps corresponding to repressive interactions between different pathways. These are marked by overlaps between a set of genes downregulated by factor "X" (i.e., interferon gamma) and another set of genes upregulated by factor "Y" (MYC oncogene). Among the total of 7,419 significant overlaps identified, 1,369 (18.4%) belong to this category (up-down). For comparison, 2,762 (37.2%) overlaps are explicitly in the same direction (i.e., up-up or down-down).

Besides the IFNG and MYC, several examples are discussed in previous sections (See Table 3 for a full list). These include the overlap between P53_Genes_All and Zeller_MYC_Dn, which is supported by the fact that p53 represses the MYC oncogene [36]. Additional file 4 includes many repressive overlaps not discussed. One of the very significant repressive overlaps, for example, is between Alzheimers_Disease_Dn and StemCell_Neural_Up. There are 276 genes that were found to be downregulated in Alzheimer's disease but were upregulated in neural stem cells. Detailed GO analysis shows that these genes are enriched with ubiquitin-dependent protein catabolic process (P < 10^-7^). This is consistent with the notion that Alzheimer's disease is one of disorders related to ubiquitin protein catabolic process [37].

The prevalence of repressive interactions among various molecular pathways highlights the complexity of cellular control machinery. This result also suggests the necessity of paying close attention to the downregulated genes and cross-checking them with upregulated genes in other situations.

## Discussion

The highly connected nature of the 1,186 gene sets is surprising. An average gene set overlaps with more than seven gene sets, above a significant level of FDR < 0.001. Moreover, the majority (80%) of the 1,186 gene sets are connected directly or indirectly as one big network. In other words, any newly defined gene sets will have approximately an 80% chance of having at least one significant overlap with a gene set in MSigDB database. Our results suggest that many seemingly unrelated stimuli/perturbations may activate or deactivate the same molecular pathways. We have discussed several unexpected overlaps in our paper while analyzing sub-networks in previous sections. One example is the shared genes among MYC target genes, serum stimulation, and interferon gamma over-expression. Our data-driven analysis confirms the connection between them: serum stimulation and interferon gamma up- and down-regulates MYC target genes, respectively.

The observation that most of the gene sets are connected to one dominant network can be explained in several ways. Researchers might be biased and focus on a small set of essential processes in cells, which would give rise to a connected network. Similarly, the MSigDB could have been selectively compiled. Another explanation for the observation is that cells respond to diverse perturbations with overlapping genes, resulting in the observed heavily connected networks. This explains the MYC pathway involvement in response to diverse stimuli. We believe that all of these factors contribute to the connectivity of the network.

An implication from this finding is to compare new gene lists obtained from genomics studies to big databases of previously published gene sets. Interpretation of gene lists remains a challenge in high-throughput genomics studies. Algorithms and databases are available and can be used to detect overrepresented genes belonging to the same pathway, GO category, target genes of transcription factors, etc. Alternatively, new gene lists can be compared with all published gene lists. Our analysis showed that very different biological processes can share a gene expression signature. Comparison with thousands of published gene sets will help in the interpretation of new gene lists, with the contextual molecular perturbation map. This is indeed similar to queries of nucleic acid sequence databases for the annotation of new sequence entries. MSigDB already has a user-friendly interface that enables users to upload their gene lists and compare them with all archived gene sets.

One of the drawbacks of this study is that we used gene sets from both human and mouse studies, and comparisons within the same species often involved different types of tissues or even cell lines. We included as many gene sets as possible based on the rationale that a) overlaps between divergent molecular pathways in these species/tissues would not be detected and b) significant overlaps, once detected, would suggest conserved molecular mechanisms across species/tissues. There are some evidence based on studies of yeast [38] and bacteria [39] suggesting that gene regulatory networks are remarkably flexible, and large scale rewiring is possible. Another limitation of this study is that our results, the highly connected modules of gene lists, were mainly validated through speculative discussions based on literature. We discussed only a subset of the modules that we deemed interesting. Two additional sub-networks related to p53 signalling and cell differentiation are discussed in Additional File 1. Further study is clearly needed to verify the identified links between diverse biological perturbations.

## Conclusions

Despite the fact that DNA microarray studies might be inconsistent across laboratories [40], we identified thousands of statistically significant overlaps between published gene lists. Summarized as a molecular signature map, our results provide key insights into underlying connections of diverse perturbations. We have found evidence that the molecular signature map is 1) highly interconnected, suggesting that overlapping sets of genes are used over and over again by cells to respond to various stimuli, and 2) modularly organized, suggesting that different responses are coordinated via functional modules.

## Methods

### Data source

We downloaded "C2" gene set files (v2.5 updated April 7, 2008) of the MSigDB [10] that contain 1,186 gene sets that represent chemical and genetic perturbations manually extracted from publications. This database also includes gene sets contributed by individual researchers and other similar databases such as the List of List Annotated (L2L) database [8].

### Statistical and network analyses

We developed a set of Perl scripts to analyze the original gene set database and evaluate the overlapping genes between all pairs. The P value for determining the significance of overlaps between two gene sets is calculated based on the hypergeometric distribution using the statistical computing software R (http://www.r-project.org). The original P values are then converted into false discovery rate (FDR) [12]. Overlaps with FDR < 0.001 were considered significant. Our approach is similar to the method used by Newman and Weiner, except that they used binominal distribution to approximate the hypergeometric distribution for faster calculation [41].

We used undirected graphs to represent the overlapping information across thousands of gene sets. A significant overlap defines an edge between the two nodes that represent the gene sets. In the network file, each edge has properties representing the number of common genes, names of the common genes and FDR value. Each node has a name, a one-sentence description and the entire gene set. The network file, available as Additional File 3, thus includes a comprehensive account for all "C2" gene sets in MSigDB. The network is visualized using Cytoscape software version 2.6.3 [42], and highly interconnected sub-networks were identified using MCODE version1.3 [15] with default settings.

To identify statistically enriched GO terms we selected the top 70 most frequently appearing genes in each sub-network and analyzed these gene lists with the DAVID web site (http://david.abcc.ncifcrf.gov/) [43,44]. If the number of genes shared by gene sets was smaller than 70, only the genes that appeared at least twice were used. The most significant terms for all GO biological process terms are listed in Table 2.

### DNA microarray data analysis

The DNA microarray dataset (Affymetrix .CEL files) of glutamine starvation [29] was downloaded from the homepage of the research group (http://jura.wi.mit.edu/sabatini_public/rapachip2/frameset1.html). The data were re-analyzed using an RMA algorithm. Genes were ranked by average fold change over 12 hours and 24 hours of glutamine starvation compared to normal control. The ranked gene sets were used for pathway analysis with the GSEA algorithm [10].

## Authors' contributions

SXG conducted the study and wrote the manuscript.

## Supplementary Material

Additional File 1 Supplementary figures and table. Click here for file

Additional File 2 **Perl script used to identify overlaps between gene sets. **This program will accept a file in *.GMT format, in which each line defines a gene set. Click here for file

Additional File 3 **A molecular signature map. **This is a file to be opened by Cytoscape. It contains a large network representing the significant overlaps between diverse molecular signatures. Click here for file

Additional File 4 **List of overlapping gene lists. **This is an Excel file that contains lists of significant overlaps between gene sets. Repressive overlaps are identified as "rep" in the third column. Click here for file

Additional File 5 Genes shared by multiple gene sets in sub-networks. Click here for file
