# Supplementary material for: Large-scale analysis of expression signatures reveals hidden links among diverse cellular processes
Source: BMC Syst Biol. 2011 May 29;5:87. doi: 10.1186/1752-0509-5-87 (PMC3123203; doi:10.1186/1752-0509-5-87)
Supplement: Additional File 1 — Supplementary figures and table. [file 1752-0509-5-87-S1.DOC]

# Supplementary document for:

# Large-scale analysis of expression signatures reveals hidden links among diverse cellular processes

### Steven X. Ge^1 §^

^1^ Department of Mathematics and Statistics, South Dakota State University, Brookings, SD 57006, USA

^§^Corresponding author

Figure S1. Distribution of the size of 1,186 gene sets in MSigDB: “C2: chemical and genetics perturbation” category.


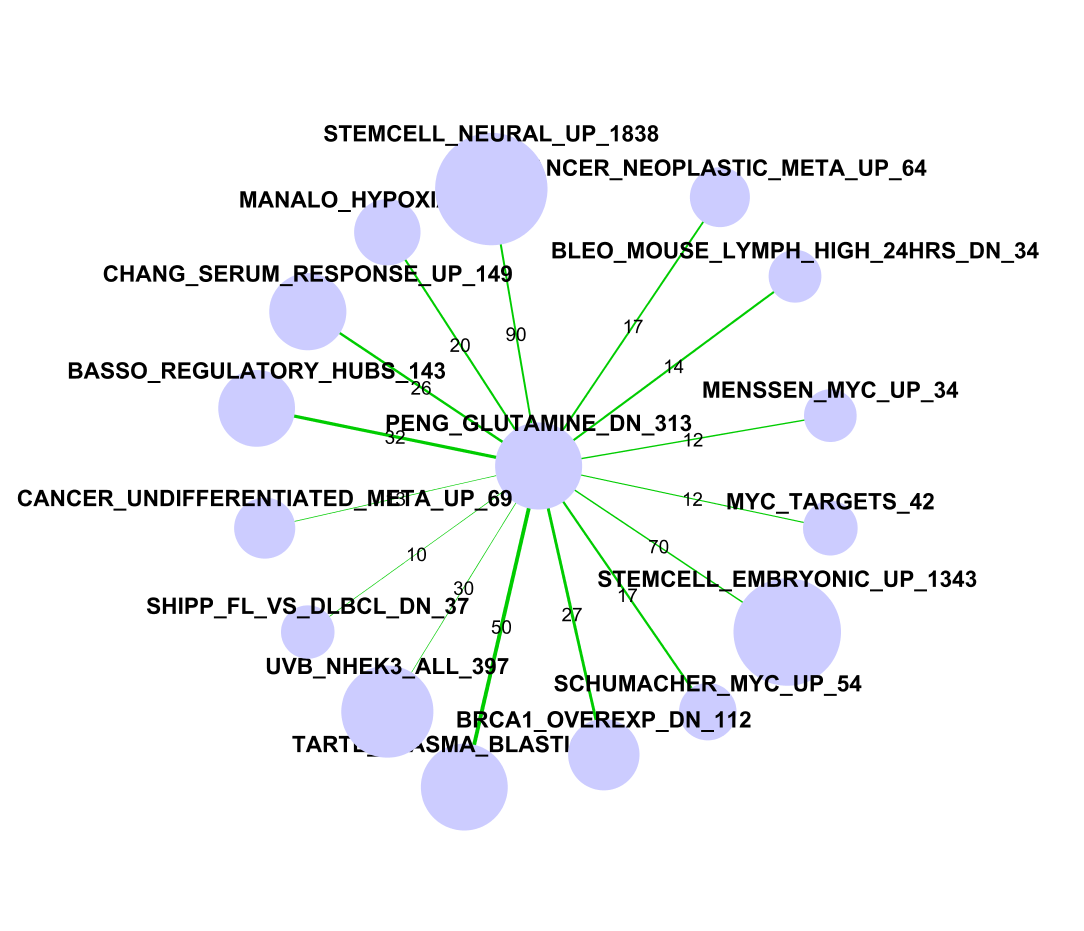


Figure S2. First neighbors of Peng_Glutamine_Dn with FDR <1e-6 in the entire network. Many MYC-related gene sets overlap significantly with this set.


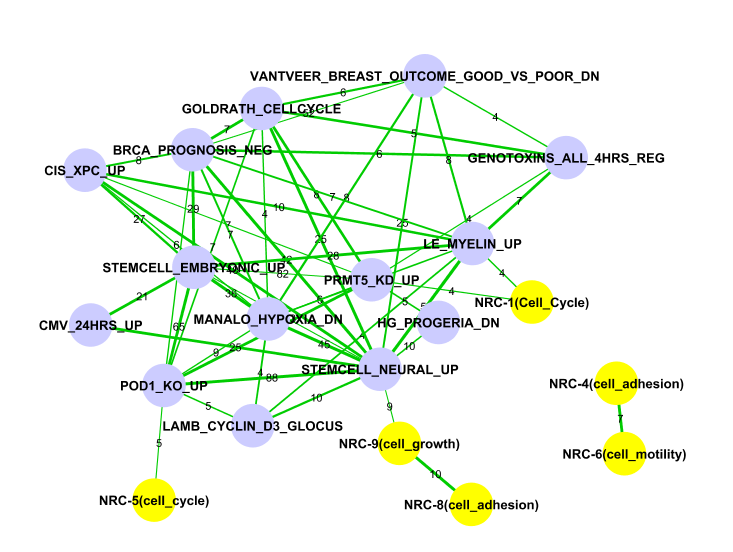


Figure S3. Comparing recently defined breast cancer gene signature with our sub-network of stem-cell and breast cancer prognosis related gene sets. Nine gene sets (NRC-1 to NRC-9) that were identified as high-quality cancer prognostic markers by Li *et al.* were compared to the gene sets shown in Fig. 4 in the main text. Three of the NRC gene sets overlap with gene sets in this subnetwork with unadjusted P value < 10^-4^. Many of these overlapped genes are cell cycle related. In addition, we identified very significant overlaps between two pairs of NRC gene sets (NRC-9 with NRC-8, NRC-4 with NRC-6).

## Multiple functions of p53 and its relationship with BRCA1 and YY1

Another important sub-network of 18 gene sets shown in Fig. S4 is related to tumor protein p53 (TP53). This sub-network is based on a small three node sub-network that is not listed in Table 1. The network shown in Fig. 5 is defined by re-running MCODE with different parameters to encourage big sub-networks. The “p53genes_all” is a set of 17 target genes of TP53 identified by using an in vivo system that tests an inducible p53’s binding to P53 responsive elements in promoter sequences of 26 genes . “Kannan_p53_up” contains 40 genes that were found to be up-regulated by p53 in a human lung cancer cell line through gene expression profiling . These genes are considered primary p53 targets, as protein synthesis was inhibited by cycloheximide. The “p53_BRCA1_up” gene set consists of genes up-regulated by p53 in mouse embryonic fibroblasts (MEFs) lacking both p53 and BRCA1 . Most of these genes are further upregulated by BRCA1. Another p53 related gene set “Stress_p53_specific_up” includes genes responsive to DNA-damaging treatments. This set significantly overlaps (q value <1x10^-5^) with the three gene sets mentioned above on several key genes, including CCNG1, CDKN1A, MDM2 and PHLDA3. But MCODE software failed to include this set in this sub-network. The “Bleo_Human_Lymph_high_4hrs_up” is identified by treating human lymphocytes with a high dosage of bleomycin, an oxidative mutagen . The original authors noted that this treatment triggers p53 target genes that are involved in DNA repair and cell cycle regulation . Therefore, many core gene sets in Fig. 6 are p53 related.


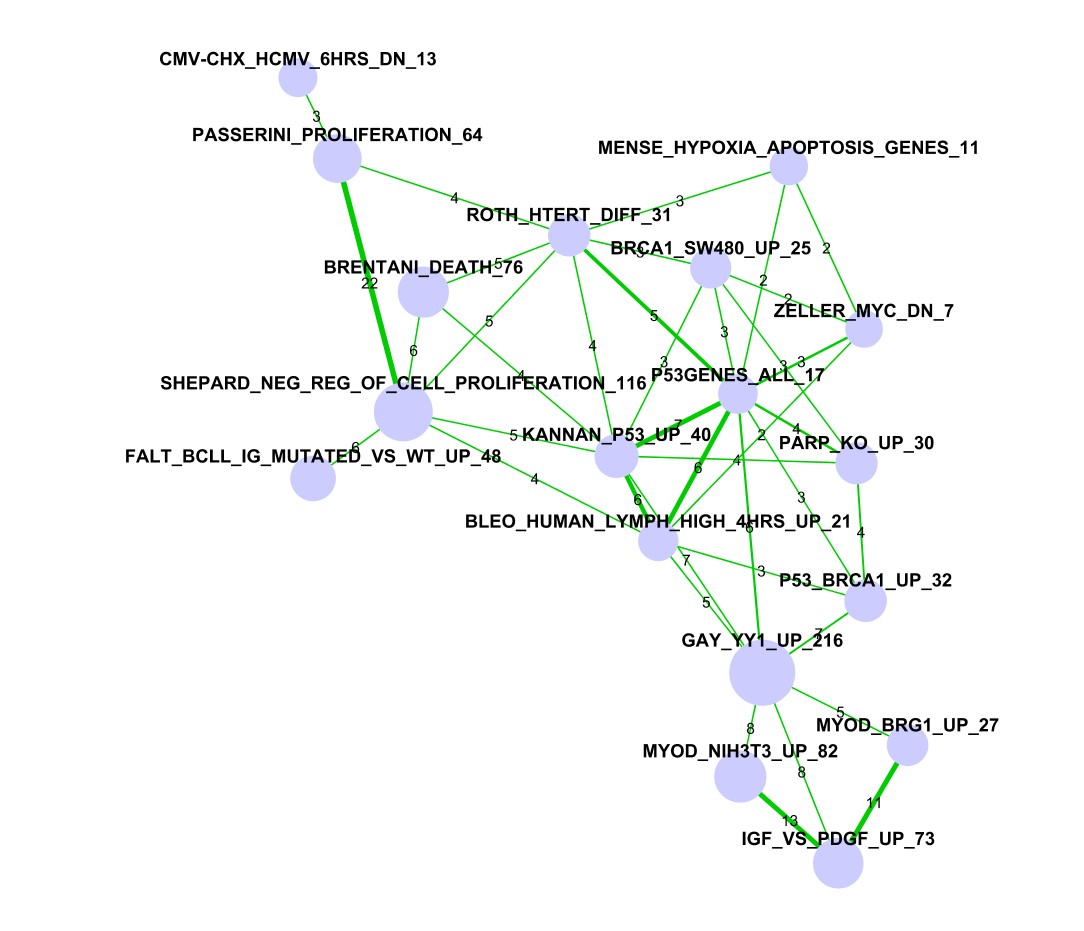


Figure S4. p53 related gene sets and their neighbours.

Our analysis also reveals interesting overlaps between p53-related gene sets and gene sets derived from other perturbations. Some of these links confirm the multiple roles p53 plays. As shown in Fig. 6, we detected significant overlapping of p53 genes with hypoxia-induced apoptosis genes, cancer related genes involved in cell death, and genes related to negative regulation of cell proliferation. The overlap between genes upregulated by p53 and genes downregulated by MYC “Zeller_MYC_DN” is another example of repressive interaction, which is in agreement with the finding that p53 represses the oncogene Myc, possibly through induction of microRNA-145 .

Also overlapping with p53 genes is “PARP_KO_UP” , a set of genes upregulated in MEFs after knockout of Poly(ADP-ribose) polymerase (PARP). The depletion of PARP enzyme leads to increased genomic instability, and results in altered expression of p53 . Thus, the common genes (CDKN1A, IGFBP6, MDM2, and PCNA) might be involved in DNA repair related functions.

Additionally, the “BRCA1_SW480_up” gene list contains genes upregulated by ectopic expression of BRCA1 through infection of human colon adenocarcinoma cells (SW480) by an adenovirus vector . Commonality between this gene set and p53 related genes indicates these two proteins can regulate many of the same set of genes independently . Finally, the gene set “Gay_YY1_up” contains genes with higher MEFs expression: ~25% of Yin Yang 1 (YY1) compared to wild type . Here, these genes should be considered as YY1 downregulated genes. The commonality between target genes of p53 and YY1 has been noticed and it is well-established that YY1 inhibits the activation of p53 . Overall, we observed that the network in Fig. 6 highlights the multiple roles of p53. The synergistic interaction of p53 with BRCA1 as well as the suppressive interaction of p53 with YY1 and MYC are confirmed.

## Connection between cell differentiation and cancer progression

The sub-network #1a in Table 1 is shown in Fig. S5. The “IDX_TSA_UP_Cluster3” includes a subset of genes upregulated during TSA-induced differentiation of fibroblasts into adipocytes . The list “Serum_Fibroblast_CellCycle” is a set of cell cycle related genes regulated by serum exposure in a variety of human fibroblast cell lines . Serum exposure can regulate the cell cycle of fibroblasts in a quasi-synchronous fashion . These two gene sets overlap with “Cancer_undifferentiated_Meta_up,” which contains 69 genes commonly upregulated in undifferentiated cancer relative to well-differentiated cancer, based on a meta-analysis of 40 published cancer related microarray studies (Rhodes et al., 2004). This set correlates with “BRCA_ER_NEG,” which contains genes highly expressed in more aggressive estrogen receptor negative (ER-) tumors . The “Li_Fetal_vs_WT_kidney_DN” gene set represents genes highly expressed in Wilms' tumor (WT) compared with fetal kidneys . It is known that WT is characterized by arrested cellular differentiation. The “DOX_Resist_Gastric_up” gene set contains 44 genes highly expressed in gastric cell lines that have developed anticancer drug resistance compared with non-resistant cells . It is remarkable that 26 of the 44 genes are upregulated in fibroblasts upon serum exposure. The significance levels of other overlaps in this sub-network are also extremely high. Broadly, the overlaps in Fig. 6 show a common set of cell cycle related genes that are highly expressed in both the normal differentiation process and in aggressive tumor cells.


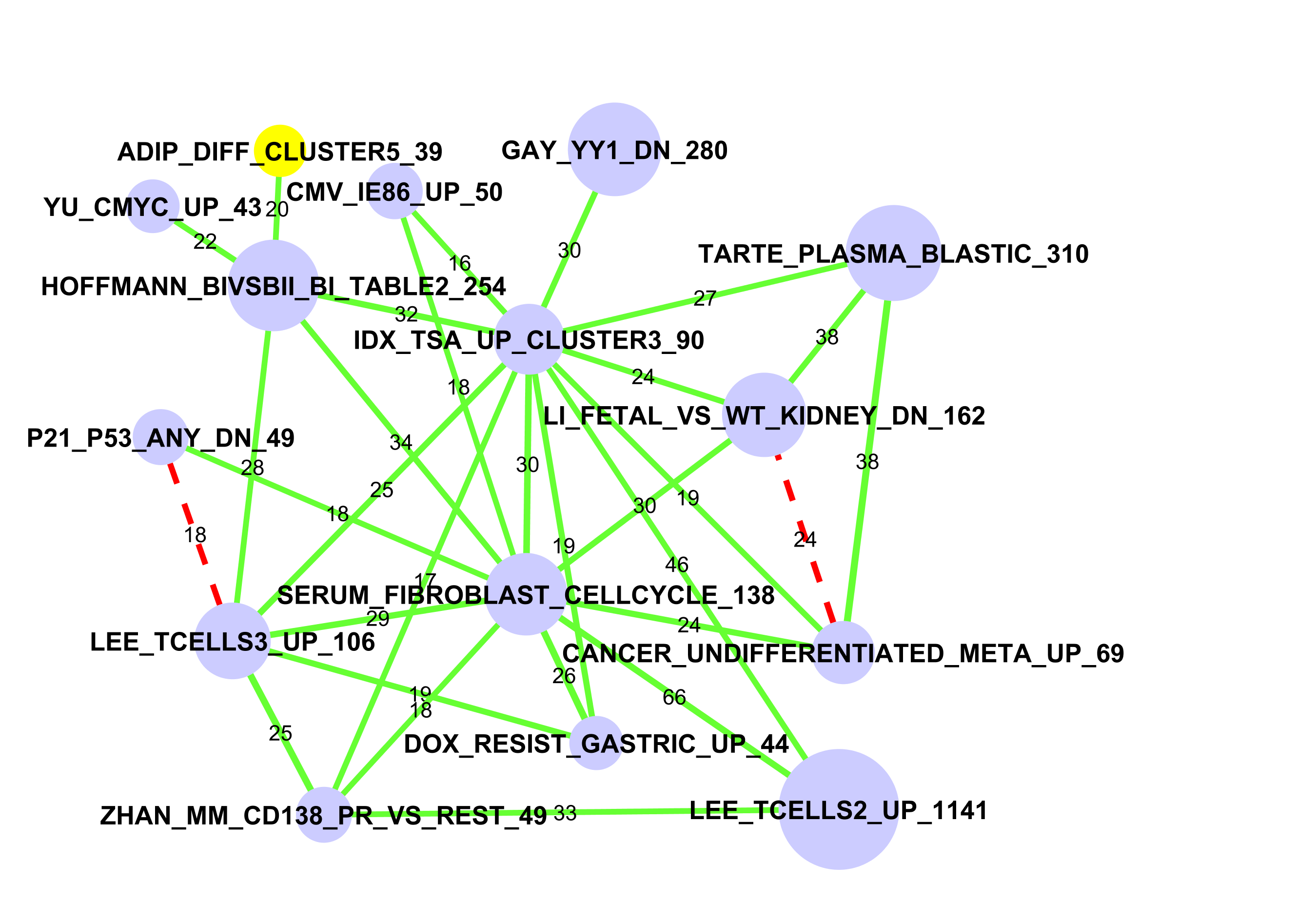


Figure S5. Sub-network #1a: Connecting cell differentiation and cancer. Only highly significant edges with FDR<1.0×10^-20^ are shown.

Table S1. Downregulated pathways identified by GSEA analysis of DNA microarray data of glutamine starvation. The pathways are ranked by Normalized Enrichment Score (NES).

# References

Reference List

1. Inga A, Storici F, Darden TA, Resnick MA: **Differential transactivation by the p53 transcription factor is highly dependent on p53 level and promoter target sequence**. *Mol Cell Biol* 2002, **22**(24):8612-8625.

2. Kannan K, Amariglio N, Rechavi G, Jakob-Hirsch J, Kela I, Kaminski N, Getz G, Domany E, Givol D: **DNA microarrays identification of primary and secondary target genes regulated by p53**. *Oncogene* 2001, **20**(18):2225-2234.

3. Ongusaha PP, Ouchi T, Kim KT, Nytko E, Kwak JC, Duda RB, Deng CX, Lee SW: **BRCA1 shifts p53-mediated cellular outcomes towards irreversible growth arrest**. *Oncogene* 2003, **22**(24):3749-3758.

4. Amundson SA, Do KT, Vinikoor L, Koch-Paiz CA, Bittner ML, Trent JM, Meltzer P, Fornace AJ, Jr.: **Stress-specific signatures: expression profiling of p53 wild-type and -null human cells**. *Oncogene* 2005, **24**(28):4572-4579.

5. Islaih M, Li B, Kadura IA, Reid-Hubbard JL, Deahl JT, Altizer JL, Watson DE, Newton RK: **Comparison of gene expression changes induced in mouse and human cells treated with direct-acting mutagens**. *Environ Mol Mutagen* 2004, **44**(5):401-419.

6. Sachdeva M, Zhu S, Wu F, Wu H, Walia V, Kumar S, Elble R, Watabe K, Mo YY: **p53 represses c-Myc through induction of the tumor suppressor miR-145**. *Proc Natl Acad Sci U S A* 2009, **106**(9):3207-3212.

7. Simbulan-Rosenthal CM, Ly DH, Rosenthal DS, Konopka G, Luo R, Wang ZQ, Schultz PG, Smulson ME: **Misregulation of gene expression in primary fibroblasts lacking poly(ADP-ribose) polymerase**. *Proc Natl Acad Sci U S A* 2000, **97**(21):11274-11279.

8. Simbulan-Rosenthal CM, Haddad BR, Rosenthal DS, Weaver Z, Coleman A, Luo R, Young HM, Wang ZQ, Ried T, Smulson ME: **Chromosomal aberrations in PARP(-/-) mice: genome stabilization in immortalized cells by reintroduction of poly(ADP-ribose) polymerase cDNA**. *Proc Natl Acad Sci U S A* 1999, **96**(23):13191-13196.

9. MacLachlan TK, Somasundaram K, Sgagias M, Shifman Y, Muschel RJ, Cowan KH, El-Deiry WS: **BRCA1 effects on the cell cycle and the DNA damage response are linked to altered gene expression**. *J Biol Chem* 2000, **275**(4):2777-2785.

10. Affar el B, Gay F, Shi Y, Liu H, Huarte M, Wu S, Collins T, Li E: **Essential dosage-dependent functions of the transcription factor yin yang 1 in late embryonic development and cell cycle progression**. *Mol Cell Biol* 2006, **26**(9):3565-3581.

11. Gronroos E, Terentiev AA, Punga T, Ericsson J: **YY1 inhibits the activation of the p53 tumor suppressor in response to genotoxic stress**. *Proc Natl Acad Sci U S A* 2004, **101**(33):12165-12170.

12. Burton GR, Nagarajan R, Peterson CA, McGehee RE, Jr.: **Microarray analysis of differentiation-specific gene expression during 3T3-L1 adipogenesis**. *Gene* 2004, **329**:167-185.

13. Chang HY, Sneddon JB, Alizadeh AA, Sood R, West RB, Montgomery K, Chi JT, van de Rijn M, Botstein D, Brown PO: **Gene expression signature of fibroblast serum response predicts human cancer progression: similarities between tumors and wounds**. *PLoS Biol* 2004, **2**(2):E7.

14. Brooks RF: **Regulation of fibroblast cell cycle by serum**. *Nature* 1976, **260**(5548):248-250.

15. van 't Veer LJ, Dai H, van de Vijver MJ, He YD, Hart AA, Mao M, Peterse HL, van der Kooy K, Marton MJ, Witteveen AT *et al*: **Gene expression profiling predicts clinical outcome of breast cancer**. *Nature* 2002, **415**(6871):530-536.

16. Li CM, Guo M, Borczuk A, Powell CA, Wei M, Thaker HM, Friedman R, Klein U, Tycko B: **Gene expression in Wilms' tumor mimics the earliest committed stage in the metanephric mesenchymal-epithelial transition**. *Am J Pathol* 2002, **160**(6):2181-2190.

17. Kang HC, Kim IJ, Park JH, Shin Y, Ku JL, Jung MS, Yoo BC, Kim HK, Park JG: **Identification of genes with differential expression in acquired drug-resistant gastric cancer cells using high-density oligonucleotide microarrays**. *Clin Cancer Res* 2004, **10**(1 Pt 1):272-284.

18. Li J, Lenferink AE, Deng Y, Collins C, Cui Q, Purisima EO, O'Connor-McCourt MD, Wang E: **Identification of high-quality cancer prognostic markers and metastasis network modules**. *Nat Commun* 2010, **1**:34.

# 
